# Pre-treatment EMG can be used to model post-treatment muscle coordination during walking in children with cerebral palsy

**DOI:** 10.1371/journal.pone.0228851

**Published:** 2020-02-12

**Authors:** Lorenzo Pitto, Sam van Rossom, Kaat Desloovere, Guy Molenaers, Catherine Huenaerts, Friedl De Groote, Ilse Jonkers

**Affiliations:** 1 Department of Movement Sciences, KU Leuven, Leuven, Belgium; 2 Department of Rehabilitation Sciences, KU Leuven, Leuven, Belgium; 3 Clinical Motion Analysis Laboratory, University Hospital Leuven, Leuven, Belgium; 4 Department of Development and Regeneration, University Hospital Leuven, Leuven, Belgium; West Virginia University, UNITED STATES

## Abstract

When treating children with Cerebral Palsy (CP), computational simulations based on musculoskeletal models have a great potential in assisting the clinical decision-making process towards the most promising treatments. In particular, predictive simulations could be used to predict and compare the functional outcome of a series of candidate interventions. In order to be able to benefit from these predictive simulations however, it is important to know how much information about the post-treatment patient’s motor control could be gathered from data available before the intervention. Within this paper, we quantified how much of the muscle activity measured after a treatment could be explained by subject-specific muscle synergies computed from EMG data collected before the intervention. We also investigated whether generic synergies could be used, in case no EMG data is available when running predictive simulations, to reproduce both pre- and post-treatment muscle activity in children with CP. Subject-specific synergies proved to be a good indicator of the patient’s post-treatment motor control, explaining on average more than 85% of the post-treatment muscle activity, compared to an average of 94% when applied to the original pre-treatment data. Generic synergies explained 84% of the pre-treatment and 83% of the post-treatment muscle activity on average, but performed relatively well for patients with low selective motor control and poorly in patients with more selectivity. Our results suggest that subject-specific muscle synergies computed from pre-treatment EMG data could be used with confidence to represent the post-treatment motor control of children with CP during walking. In addition, when performing simulations involving patients with a low selective motor control, generic synergies could be a valid alternative.

## Introduction

Cerebral palsy (CP) is caused by a lesion in the developing brain and leads to a variety of movement disorders. The primary lesion itself is not progressive, but secondary musculoskeletal pathologies often worsen over time, thereby increasingly affecting mobility [1–3]. To improve motor performance or to prevent further deterioration, several orthopedic and non-orthopedic treatments are available [4–7]. The selection of the appropriate treatment, however, is a challenging task, which does not always achieve the desired outcome [8]. While the use of three-dimensional gait analysis has proven to be successful in reducing the rate of unsuccessful treatments that require follow-up surgeries [9], musculoskeletal models and computational simulations can provide further help in selecting the most promising treatment. In particular, they would allow performing in-silico evaluation and comparison of the effects of different therapeutic interventions on the patient’s condition [10–12]. Such predictive simulations are capable of generating a motion pattern without relying on any previous knowledge about such motion. Only information defining the musculoskeletal system and motor control strategy are needed [13] [14]. When applied to subjects with neurological impairments these simulations are particularly sensitive to the motor control model used [14,15]. Thus, it is important to generate a model of the patient’s motor control that is representative of the specific impairment. It is also important to know how representative of the post-operative condition a model built using only pre-operative data is, since this scenario would be the most helpful in clinical practice.

Activations from a large number of muscles, usually collected using surface EMG, can be accurately described by a small number of independent modules, also known as muscle synergies [16–19], which are a common way to summarize features of a subject’s motor control. Compared to healthy subjects, patients with neurological disorders display altered sets of synergies [20]. In particular, patients with CP often exhibit a lower number of synergies, indicative of reduced selective motor control [21]. Synergies are thought to reflect the neural architecture of the patient [18,22] at levels of the brain stem and the spinal cord [23,24] and they have proven to be resilient to changes in kinematics and kinetics [25]. In this perspective, synergies can be expected to remain unaffected by most orthopedic treatments, as these do not directly affect the central nervous system. Despite the invasive nature of the treatments administered to patients with CP, EMG signals, and therefore synergies, indeed appear not to change after treatment [26] or only minimally[27].

The current study, *firstly* investigates if subject-specific muscle synergies computed before a treatment can explain post-treatment muscle activations, thereby assessing the extent of motor control adaptations following the treatment. While muscle synergies seem to undergo little changes after treatment, from a modelling perspective, the amount of post-treatment muscle activity that can be explained by a model of motor control based only on information collected before the treatment is still an open question. We investigated this for patients treated with botulinum toxin injections (BOTOX) and single-event multilevel surgeries (SEMLS). In this way, we evaluated a treatment that mainly affects muscle physiology, and one that more radically affects the musculoskeletal architecture. We explored two different approaches to reconstruct post-treatment activations with pre-treatment synergies, reflecting two possible mechanisms of motor control adaptation [28]. One approach allows variations in the timing of the synergy activations (activation optimization approach—AOA) and the other allows changes in their composition (weight optimization approach–WOA). Furthermore, we investigated if subject-specific synergies provide more accurate reconstructions of the pathological muscle activations than of the activations from typically developed (TD) children. *Secondly*, we investigated if generic synergies could be used as alternative to subject-specific synergies providing a comparable degree of accuracy in EMG reconstruction. For both these aims, we analyzed how well individual muscle activity could be represented when constructed using either subject-specific pre-treatment or generic synergies. *Finally*, we identified pre-treatment factors that determine the changes in post-treatment motor control or the performance of the EMG reconstruction methods using subject-specific synergies.

We hypothesized that the motor control of the patients would not change significantly after the treatment, thus allowing pre-treatment synergies to explain most of the post-treatment EMG signals. We further hypothesized that pre-treatment synergies capture the pathological features of the subjects’ motor control and therefore are unable to explain EMG signals from TD children with unimpaired motor control. If the proposed reconstruction methods prove to adequately reproduce the post-treatment activations and to distinguish between impaired and normal motor control, it would mean that muscle synergies are able to model the motor control of the patient and that they could be used with confidence in computational simulations trying to predict the post-treatment condition of patients with CP.

## Materials and methods

This study was carried out in accordance with the recommendations of the local ethical committee [Commissie Medische Ethiek KU Leuven (Medical Ethics Committee UZ KU Leuven/Research)]. In accordance with the Declaration of Helsinki, written informed consent was obtained of the participants’ parents prior to the experiment. The participants’ parents supervised the measurement session. The protocol was approved by the Medical Ethics Committee UZ KU Leuven/Research.

We analyzed retrospective three-dimensional motion capture data collected from 46 children with diplegic CP (25 were treated with BOTOX and 21 underwent SEMLS) and from seven TD children. The patients included in the study were able to walk independently and had GMFCS I or II. None of them had received major surgeries prior to the study, nor BOTOX injections in the previous 6 months. More details about the condition of the patients and the administered treatments are included in S1 Appendix. In participants with CP, gait analysis data was collected before and after the treatment (Table 1). On average, the collection of post-treatment data from SEMLS patients took place later in time than for BOTOX patients (on average 601 vs 112 days, respectively). This difference is due to the more invasive nature of SEMLS, requiring a longer rehabilitation. Spasticity, strength and selectivity scores were collected during a standardized clinical examination prior to the treatment [29]. The output of this examination consisted of a list of values, corresponding to the assessment of different joints in different poses and under different conditions. For this analysis, we averaged the different entries for each leg.

**Table 1:** Demographics of the participants of the study. Values are reported as mean (standard deviation). GPS_PRE_ stands for pre-treatment gait profile score, with higher scores indicating more deviation from normal kinematics.

	BOTOX *(25 subjects)*	SEMLS *(21 subjects)*	TD *(7 subjects)*
**Age** (years)	8.3 (2.1)	11.5 (3.1)	8.6 (2.4)
**Height** (cm)	127 (14)	138 (16)	131 (11)
**Weight** (kg)	27.0 (10.3)	34.2 (15.8)	28.7 (6.2)
**Time between observations** (days)	112 (85)	601 (216)	-
**GPS**_**PRE**_ (deg)	9.3 (2.7)	10.7 (2.5)	-

During gait analysis, the trajectories of a set of reflective markers on the lower limbs (Vicon Plug-in-Gait marker set derived from the Helen Hayes marker set [30]) were recorded at 100 Hz using a 10–15 camera motion capture system (Vicon Motion Systems, Oxford, UK) for one static trial and multiple walking trials at self-selected walking speed. Simultaneously, EMG data was collected at 2000 Hz (Zerowire, Cometa, Italy) from eight major muscles per leg (rectus femoris, vastus lateralis, biceps femoris long, medial hamstrings, tibialis anterior, gastrocnemius, soleus, and gluteus medius). Gait Profile Score (GPS) [31] was computed for both the pre- and post-treatment conditions. EMG signals were high-pass filtered at 40Hz, demeaned, rectified and low-pass filtered at 6 Hz with a 6^th^ order Butterworth filter, similar to [32]. For each participant, we analyzed data coming from each of the two legs independently. Only legs with five complete gait cycles (from heel strike to heel strike) with eight uncorrupted EMG signals in both the pre- and post-treatment conditions were considered eligible for synergy analysis.

From the EMG signals collected during the pre-treatment gait analysis of the patients, we extracted two kinds of muscle synergies, defined as subject-specific and generic synergies, and used them for reconstructing the post-treatment and TD EMG data. Muscle synergies were extracted from the EMG signals using non-negative matrix factorization (NNMF) [20]. To extract subject-specific synergies, EMG signals coming from five gait strides of a patient were normalized to 101 samples per stride and concatenated in a matrix *EMG*, with *Nm* rows and *Nc × Nt* columns, where *Nm* = 8 is the number of muscles, *Nc* = 5 is the number of gait cycles and *Nt* = 101 is the number of samples. Before running the NNMF, the EMG channels were normalized to have unitary standard deviation [33] in order to eliminate differences in amplitudes caused by electrodes placement in different sessions and in different patients. For a predefined number of synergies (*Nsyn*), NNMF populates a matrix *H* (*Nsyn* rows and *Nc × Nt* columns), defining the activation profile of each synergy, and a matrix *W* (*Nm × Nsyn*) of weight vectors, defining how much each muscle is activated by the corresponding synergy, by minimizing the difference between the input signals *EMG* and reconstructed signals *W × H*. To define the required number of synergies, we used a bootstrap procedure [34]. We resampled both the EMG signals and the H matrix consistently, using the Matlab function *datasample*. The newly created resampled matrices had the same dimensions as the originals. The time samples composing these matrices were picked randomly so that some of the original samples were excluded and others could appear multiple times. To increase the robustness in the identification of the number of synergies, we repeated this operation 500 times, and computed the variability accounted for (VAF) in each of these cases for any given number of synergies, leading to a distribution of VAF values. We increased the number of synergies until the 95^th^ percentile of this distribution was greater than 90%. We quantified VAF as [35]:

$$VAF=\frac{{\left(\sum_{t}\sum_{m}\left({EMG}_{m,t}^{xp}\times {recEMG}_{m,t}\right)\right)}^{2}}{\sum_{t}\sum_{m}{{EMG}_{m,t}^{xp}}^{2}\times \sum_{t}\sum_{m}{{recEMG}_{m,t}}^{2}}\times 100$$

Where *recEMG = W × H* is the signal reconstructed from the computed synergies and *EMG*^*xp*^ is the experimental data being reconstructed, which can be pre-treatment, post-treatment or TD data, leading to *VAF*_*pre*_, *VAF*_*post*_ and *VAF*_*TD*_, respectively. To extract generic synergies, we first divided the analyzed patients in groups according to *Nsyn*. For each of these groups, the pre-treatment EMG signals from all gait cycles of all patients were concatenated in a matrix with *Nm* rows and *Nc × Nt × Np* columns, where *Np* is the number of patients assigned to the group. Then, we extracted for each group a number of synergies equal to the respective *Nsyn* using NNMF. Subsequently, the pre-treatment muscle synergies (both subject-specific and generic) were used to reconstruct both the EMG data collected after the treatment from the same child and the EMG data of the seven TD children. For each of the patients, the values of the seven TD reconstructions are then reported averaged together. In addition, generic synergies were also used to reconstruct the pre-treatment EMG data. This is motivated by the fact that generic synergies are extracted from data collected from multiple subjects and there might be patients for which the fit of reconstruction is low. Two different ways in which synergies are altered when movement requirements change have been suggested [28]. A faster way, expressed by changes in the activation profiles of the synergies (matrix *H*) and a slower one, expressed by changes in the synergy structure defining the co-activation patterns of the muscles (matrix *W*). Based on these findings, we investigated both ways of motor control adaptation. We performed the EMG reconstruction using two different approaches. In the Activation Optimization Approach (AOA), we kept the pre-treatment weight vectors fixed and allowed the activation profiles to change to optimize the fit with the chosen experimental EMG. In the Weight Optimization Approach (WOA), we kept the activation profiles fixed and allowed the weight vectors to change to optimize the fit. In both cases, the optimization minimized the cost function:

$$\sum_{t=1}^{Nt}\sum_{m=1}^{Nm}{err}_{mt}$$

with

$$err={\left({EMG}^{xp}-W_{pre}\times H_{opt}\right)}^{2}$$

in AOA, or

$$err={\left({EMG}^{xp}-W_{opt}\times H_{pre}\right)}^{2}$$

in WOA. Where *pre* and *opt* represent the pre-treatment and optimized values, respectively. During the optimization, activations and weights were constrained to be positive. The optimization was solved using the Matlab function *fmincon*.

For both the subject-specific and generic synergies, NNMF returns a matrix *W* (size *Nm × Nsyn*) which can be directly used in the signal reconstruction optimization. The matrix *H*, however, has *Nc × Nt* columns for the subject-specific synergies and *Nc × Nt × Np* columns for the generic synergies. Therefore, to be used in the optimization, the time windows corresponding to each of the concatenated trials were averaged together, producing a single normalized activation profile of dimension *Nsyn × Nt*. We evaluated the goodness of the approach (*GOOD*) of using pre-treatment subject-specific muscle synergies to reconstruct post-treatment EMG data by comparing the VAF in the post- and pre-treatment condition (*VAF*_*PRE*_ and *VAF*_*POST*_, respectively), defined by the difference *GOOD = VAF*_*PRE*_*−VAF*_*POST*_. When *GOOD* is equal to 0%, the pre-operative muscle synergies are able to explain the post-treatment data with the same accuracy as the pre-treatment data. The goodness of using generic muscle synergies was evaluated by comparing the subject-specific *VAF*_*PRE*_, defined during NNMF, with the values of *VAF*_*PRE*_^*gen*^ and *VAF*_*POST*_^*gen*^, which are respectively the VAF of the pre- and post-treatment data with the generic synergies, thus leading to *GOOD*_*PRE*_^*gen*^ and *GOOD*_*POST*_^*gen*^. We defined the pre-treatment motor control impairment (*IMP*) as the difference between *VAF*_*PRE*_ and the average VAF of TD EMG explained by the pre-treatment synergies (*VAF*_*TD*_), therefore *IMP = VAF*_*PRE*_*−VAF*_*TD*_. A subject with *IMP* equal to 0% has synergies that explain the variability of the TD EMG as well as the variability of its own EMG and would hence have no motor control impairments. The difference *VAF*_*POST*_*−VAF*_*TD*_, defines the specificity of the reconstruction (*SPEC*). A value of *SPEC* equal to 0% means that pre-treatment synergies explain the post-treatment and TD EMG with the same accuracy, thus failing to capture the difference between healthy motor control and the individual’s motor impairment. A higher value of *SPEC* indicates a greater need to use subject-specific synergies in post-treatment computational simulations. The values of *GOOD* and *SPEC* were also computed for each of the muscles independently. Statistical comparisons of the VAF values between different groups and approaches were performed using the *ttest2* function in Matlab. The statistics on the *GOOD* and *SPEC* values, with respect to zero, was performed using the *ttest* function.

We performed a stepwise regression analysis [36,37] (Matlab function *stepwiselm*) to evaluate whether the performance of our EMG reconstruction methods, described by *GOOD* and *SPEC*, as well as changes in the patients’ synergy compositions with treatment are related to clinical data collected pre-treatment (Table 2). At each iteration, the algorithm adds or removes one of the independent variables (or their mutual interaction) from the model according to their effect on the p-value of the model fit. The dependent variables, *dW* and *dH*, describe how the synergy composition changed between the observations. To compute *dW* and *dH* we performed a NNMF on the post-treatment data using the same number of synergies defined for the pre-treatment data. To be able to compare the synergies between conditions, we identified couples of similar synergies using a clustering technique based on synergy weights. In this way, each of the pre-treatment synergies was associated to the most similar post-treatment synergy. To compute *dW*, we computed the correlation coefficient for each pair of vectors of synergy weights (e.g. a patient with four synergies has four pairs of weights vectors and four pairs of activations), these coefficients were then averaged to return one value for each patient. *dH* was quantified as the RMSE between the pre- and post-treatment activation profiles (normalized to one) and then averaged across synergies.

**Table 2:** Results from the stepwise regression analysis. For detailed explanation cfr. Matlab documentation for function ‘fitlm’. We considered the following dependent variables. **SPEC**_**W**_: specificity of reconstruction (VAF_POST_−VAF_TD_) relative to the weight optimization method, (% VAF). **GOOD**_**W**_: goodness of reconstruction (VAF_PRE_−VAF_POST_) relative to the weight optimization method, (% VAF). **SPEC**_**H**_: specificity of reconstruction relative to the activation optimization method, (% VAF). **GOOD**_**H**_: goodness of reconstruction relative to the activation optimization method, (% VAF). **dW**: change in muscle synergies vectors of weights after the treatment, (correlation coefficient between pre- and post-treatment synergy weights). **dH**: change in muscle synergies activation profiles after the treatment, (RMSE between pre- and post-treatment synergy activations). **GPS**_**diff**_: change in Gait Profile Score after the treatment, (degrees). The independent variables are the following. **tr**: Treatment administered to the patient, (BOTOX/SEMLS). **dT**: time between pre- and post-treatment gait analysis, (days). **Age**: age of the patient at the time of surgery, (years). **IMP**_**W**_: impairment of the patient measured in the pre-treatment condition. (VAF_PRE_−VAF_TD_) relative to the weight optimization method, (% VAF). **IMP**_**H**_: impairment of the patient measured in the pre-treatment condition relative to the activation optimization method, (% VAF). **SPA**: spasticity score measured in the pre-treatment clinical examination. **STR**: strength score measured in the pre-treatment clinical examination. **SEL**: selectivity score measured in the pre-treatment clinical examination. **GPS**_**pre**_: Gait Profile Score computed in the pre-treatment gait analysis, (degrees). **BOTOX**_1-5_: botulinum injection site. 1-rectus femoris, 2-biceps femoris, 3-medial hamstrings, 4-gastrocnemius, 5-soleus, (yes/no).

model	p	R^2^	adj R^2^	coefficients	estimate	SE	tStat	P
**SPEC**_**H**_ = 1 + Age + IMP_H_ + BOT1	6.5e-14	0.60	0.59	Intercept	-11.12	2.87	-3.88	2.4e-04
				Age	0.64	0.22	2.94	4.5e-03
				IMP_H_	0.81	0.08	9.67	1.8e-14
				BOT1	3.41	1.68	2.04	4.6e-02
**GOOD**_**H**_ = 1 + Age + STR	5.4e-04	0.19	0.17	Intercept	25.44	4.77	5.33	1.1e-06
				Age	-0.60	0.22	-2.66	9.7e-03
				STR	-3.07	1.22	-2.52	1.4e-02
**SPEC**_**W**_ = 1 + IMP_W_ + BOT1	3.7e-08	0.39	0.37	Intercept	-5.15	3.34	-1.54	0.13
				IMP_W_	0.81	0.14	5.91	1.1e-07
				BOT1	4.46	1.50	2.98	4.0e-03
**GOOD**_**W**_ = 1 + BOT1	4.4e-03	0.11	0.10	Intercept	9.75	0.66	14.78	2.3e-23
				BOT1	-4.44	1.51	-2.94	4.4e-03
**dW** = 1 + IMP_H_ + BOT1	1.8e-03	0.16	0.14	Intercept	0.89	5.1e-02	17.23	6.9e-27
				IMP_H_	-7.1e-03	2.4e-03	-2.90	5.0e-03
				BOT1	-0.11	4.9e-02	-2.15	3.5e-2
**dH** = 1 + IMP_H_ + BOT1	4.4e-06	0.30	0.30	Intercept	0.13	1.7e-02	7.53	1.3e-10
				IMP_H_	2.7e-03	8.1e-04	3.39	1.2e-3
				BOT1	6.6e-02	1.6e-02	4.05	1.3e-4
**GPS**_**diff**_ = 1 + tr + Age + GPS_pre_	4.2e-14	0.61	0.60	Intercept	2.44	1.55	1.56	0.12
				tr	1.63	0.44	3.68	4.5e-04
				Age	-0.16	7.1e-02	-2.28	2.5e-3
				GPS_pre_	-0.47	7.0e-02	-6.75	4.0e-09

## Results

### Subject description

We analyzed 73 legs from 46 patients from which 25 were treated with BOTOX and 21 underwent SEMLS. Pre-treatment muscle EMG of 12, 47 and 14, out of these 73 legs, could be described by two, three and four synergies, respectively. These synergies accounted on average for 94.0% (SD 1.6%) of the variability of the pre-treatment EMG data (*VAF*_*PRE*_).

**Fig 1:**
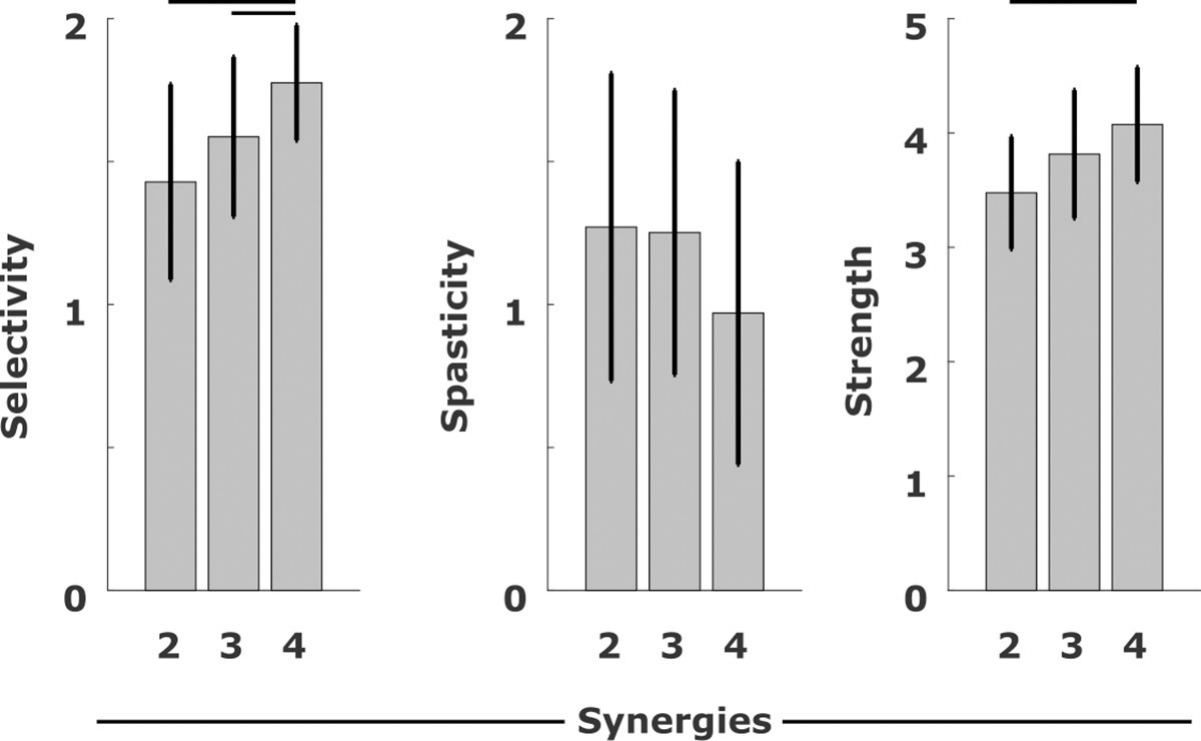
Selectivity, spasticity and strength scores. Scores for Selectivity, Spasticity and Strength from the subjects, grouped according to the number of synergies. For the Selectivity score, 2 is the maximum selectivity. For the Spasticity score, 0 means no spasticity and 2 is the maximum. For the Strength score, 5 is the maximum. Black lines indicates significant differences between two quantities (p<0.05).

Based on the pre-treatment clinical examinations, the cases assigned to the group with two and three synergies had a lower selectivity score with respect to the group with four. The cases in the group with two synergies had a lower strength score with respect to the group with four. No differences were observed for the spasticity scores (Fig 1).

### Do subject-specific synergies represent pathological, post-treatment motor control?

**Fig 2:**
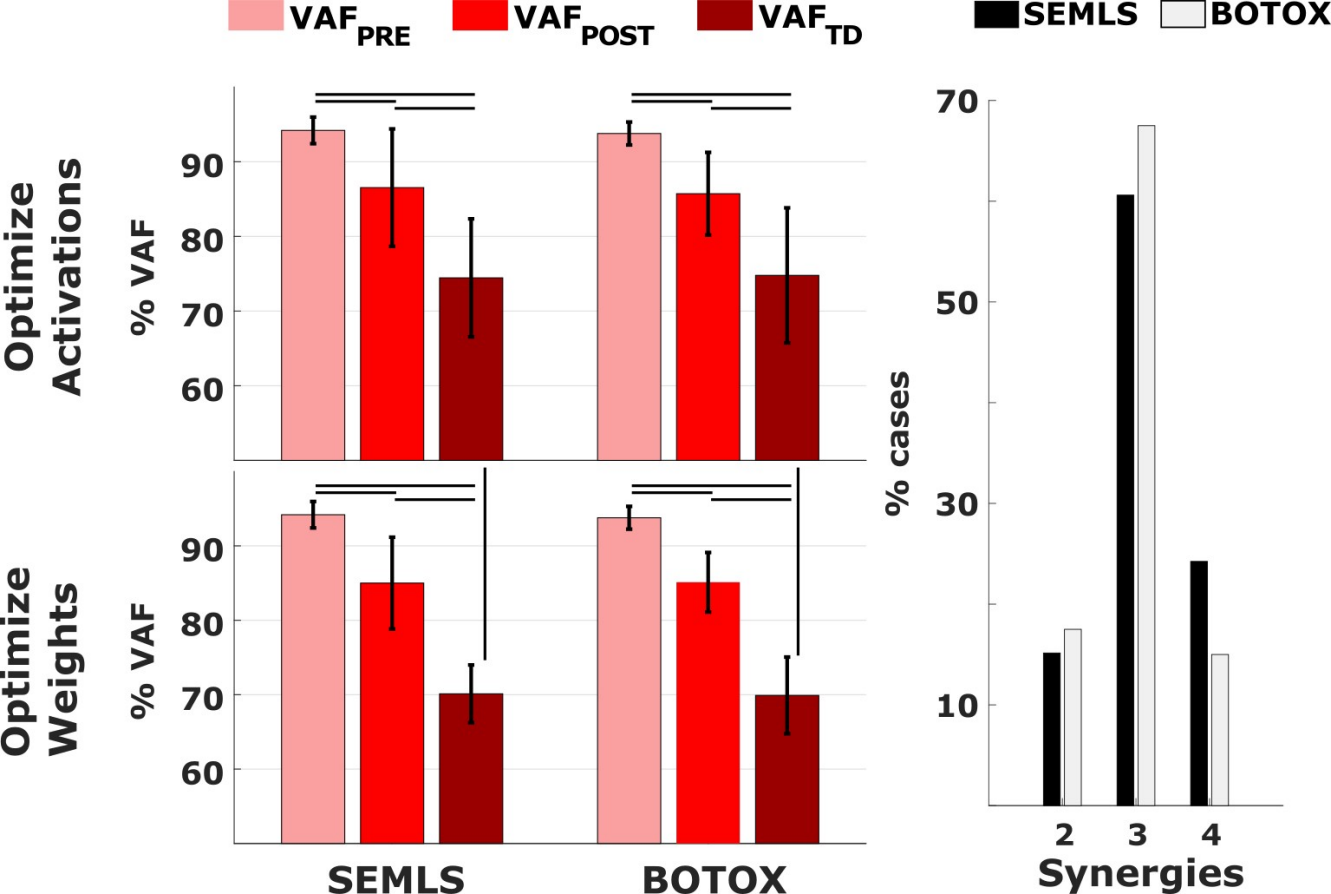
VAF values and number of synergies. On the left, mean and standard deviations for the VAF values in the PRE, POST and TD conditions, split acoording to the treatment and the reconstruction method. There were no significant differences in the VAF values between the BOTOX and SEMLS groupsBlack lines indicate significant differences (p<0.05). On the right, number of synergies explaining the pre-treatment EMG data.

We used the subject-specific synergies to reconstruct EMG data collected from the patients during their post-treatment gait analysis as well as data collected from typically developed children. Such data reconstruction was performed with two approaches. In the activation optimization approach (AOA), the vectors of synergy weights computed in the pre-treatment condition were kept fixed, and the synergy activations were optimized to best match the target data. In the weight optimization approach (WOA), synergy activations were kept fixed, and the weights optimized. The number of synergies and the variability accounted for of the post-treatment (*VAF*_*POST*_) and TD (*VAF*_*TD*_) EMG were not significantly different between the BOTOX and SEMLS populations (Fig 2). Therefore, for the remaining computations of this paper, BOTOX and SEMLS treatments were not analyzed separately. In addition, we separately analyzed results from three groups defined by the number of pre-treatment synergies.

**Fig 3:**
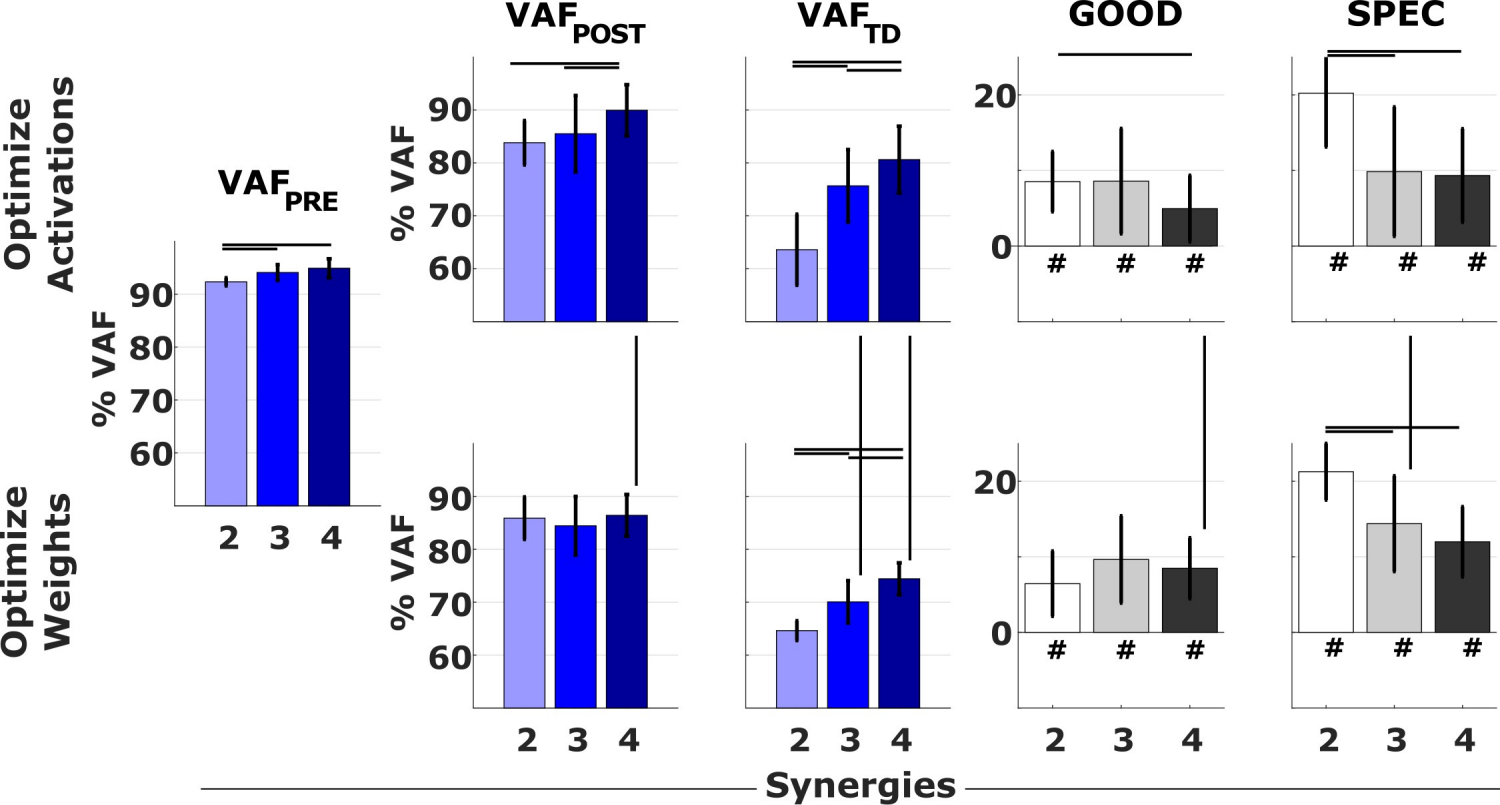
VAF when using subject–specific synergies. Results from the EMG reconstructions using pre-treatment muscle synergies. Colored bars report mean and standard deviation of the VAF values in the PRE, POST and TD conditions. Values for VAF_PRE_ are defined during synergy extraction with NNMF. Grey bars report the mean and standard deviations of the differences GOOD = VAF_PRE_−VAF_POST_ and SPEC = VAF_POST_−VAF_TD_ computed on a subject-by-subject basis. Black lines indicate significant differences (p<0.05). The symbol # indicates values of GOOD and SPEC that are significantly (p<0.05) greater than zero.

Subject-specific pre-treatment synergies largely captured the variability of the patients’ post-treatment EMG but were less successful in explaining the variability of TD EMG. Pre-treatment synergy weights (AOA) explained on average 86 ± 6.6% and 74.6 ± 8.5% of the variability of the post-treatment and TD EMG, respectively. Pre-treatment activation patterns (WOA) explained on average 85.1 ± 5.0% and 70.0 ± 4.5% of the variability of the post-treatment and TD EMG, respectively. In addition, pre-treatment synergy weights (AOA) explained more of the post-treatment EMG (higher *VAF*_*POST*_) in the group with four synergies compared to the groups with two and three synergies (Fig 3), whereas there was no difference between groups in the variability accounted for by the pre-treatment activation profiles (WOA).

In all groups and for both approaches, the subject-specific pre-treatment synergies explained on average 7.9 ± 6.2% less of the variability of the post-treatment than the pre-treatment EMG as defined by the difference *GOOD = VAF*_*PRE*_*−VAF*_*POST*_. We found little differences between groups and optimization approaches. There were no differences in the values of *GOOD* between the different groups for the WOA (6.4% ± 4.3%, 9.6% ± 5.8% and 8.5% ± 4.0% for the groups with two, three and four synergies, respectively). For the AOA, *GOOD* was higher in the group with two synergies than in the group with four synergies (values were 8.5% ± 3.9%, 8.6% ± 7.0% and 4.9% ± 4.4%, with two, three, and four synergies, respectively).

The quality of TD data reconstruction based on CP pre-treatment synergies (*VAF*_*TD*_) was lower in the groups characterized by fewer synergies, with both approaches (Fig 3). For the groups with three and four synergies, pre-treatment synergy weights better explained TD EMG than pre-treatment activation patterns (*VAF*_*TD*_ higher for AOA than for WOA), suggesting that for these patients co-activation patterns, captured by synergy weights, are more similar to TD than activation profiles.

In all groups and approaches, the pre-treatment synergies explained the post-treatment EMG better than the TD EMG and this was especially true for the group with two synergies. This difference represents the specificity of the reconstruction methods, thus *SPEC = VAF*_*POST*_*−VAF*_*TD*_. *SPEC* represents to what extent the reconstruction captures the specific features of the patient’s motor control compared to a typically developed control. Values for *SPEC* were 20.2% ± 4.2%, 9.8% ± 7.2% and 9.3% ± 4.8% for the AOA and 21.3% ± 4.0%, 14.4% ± 5.5% and 12.0% ± 3.9% for the WOA for the groups characterized by two, three and four synergies, respectively.

### Do generic synergies represent pathological motor control?

**Fig 4:**
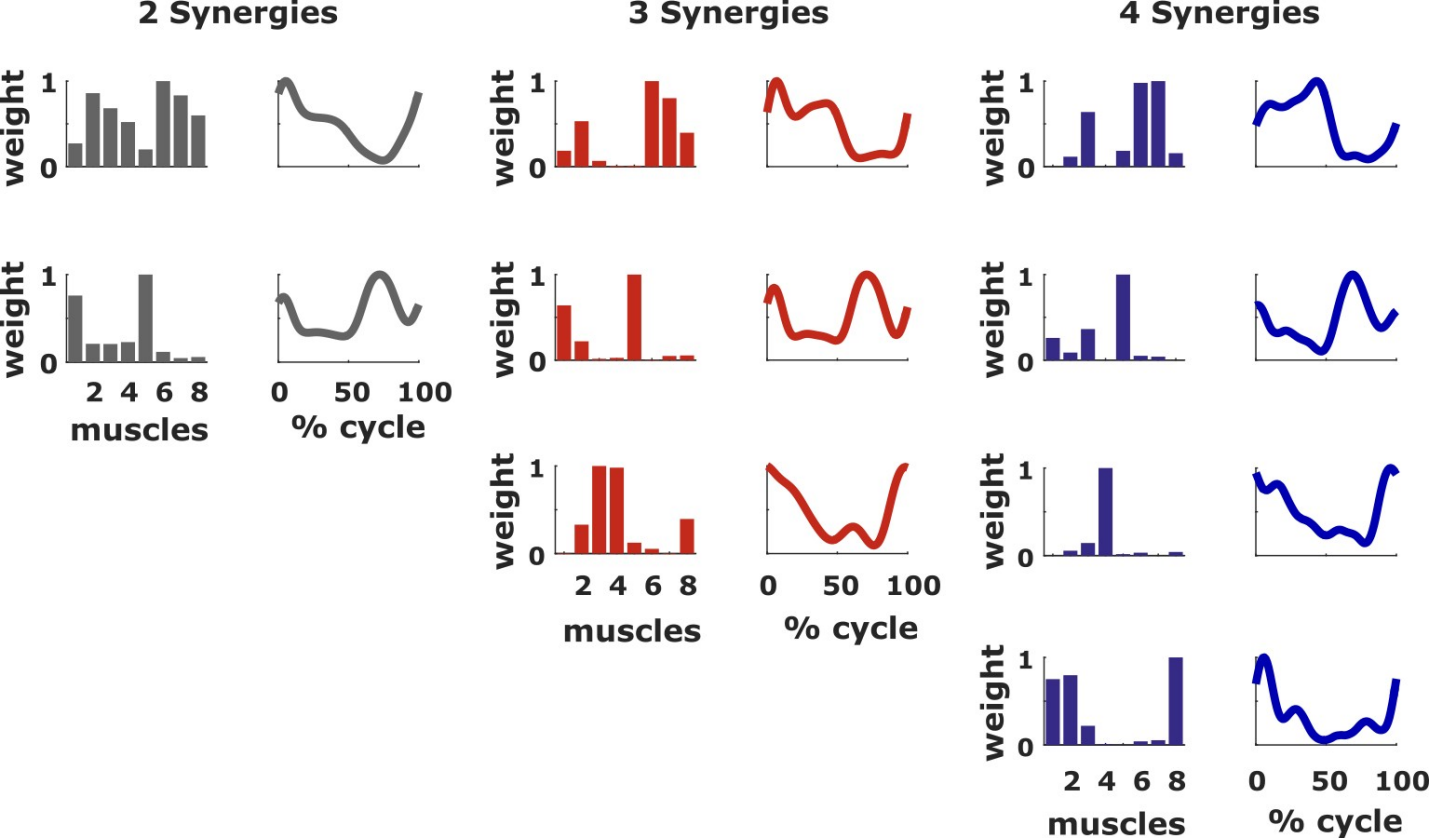
Composition of the generic synergies. Weights and activations for the extracted sets of generic synergies. Muscle names: 1 Rectus femoris, 2 Vastus lateralis, 3 Biceps femoris, 4 medial hamstrings, 5 Tibialis anterior, 6 Gastrocnemius, 7 Soleus, 8 Gluteus medius.

**Fig 5:**
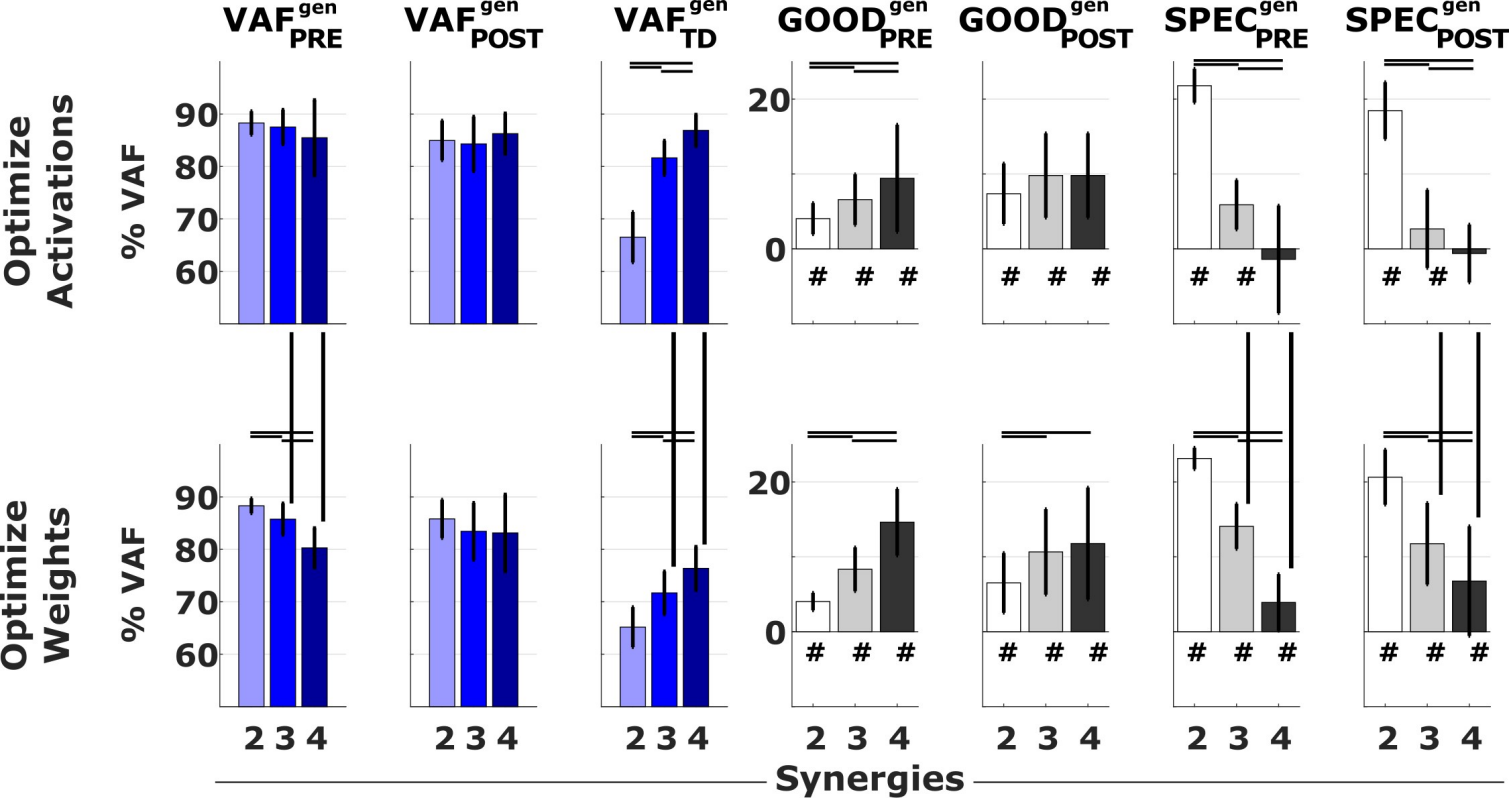
VAF when using the generic synergies. Results from the EMG reconstructions using generic synergies. Colored bars report mean and standard deviation of the VAF values in the PRE, POST and TD conditions. Grey bars report the differences GOOD_PRE_^gen^ = VAF_PRE_−VAF_PRE_^gen^, GOOD_POST_^gen^ = VAF_PRE_−VAF_POST_^gen^, SPEC_PRE_^gen^ = VAF_PRE_^gen^–VAF_TD_^gen^ and SPEC_POST_^gen^ = VAF_POST_^gen^–VAF_TD_^gen^. Black lines indicate significant differences (p<0.05). The symbol # indicates values of GOOD and SPEC that are significantly (p<0.05) greater than zero.

Generic synergies were separately determined for three groups according to the pre-treatment number of synergies (Fig 4). These were extracted from a data matrix that was obtained by concatenating all the gait cycles of all the legs within a group. These sets of synergies were successful in explaining patients’ EMG although they were less accurate than subject-specific synergies. Generic synergy weights accounted for 87.3 ± 4.2% and 84.8 ± 4.8% of the variability in pre- and post-treatment EMG respectively, whereas generic activation profiles accounted for 85.1 ± 3.9% and 83.8 ± 5.6% of the variability in the pre- and post-treatment EMG, respectively (Fig 5). When looking at the VAF of the pre-treatment data, the difference between the values obtained with the reconstructions using subject-specific synergies and generic synergies were 4.0 ± 2.1%, 6.6 ± 3.4% and 9.4 ± 7.2% for AOA and 4.0 ± 1.2%, 8.3 ± 2.9% and 14.6 ± 4.4% for WOA, for the groups of two, three and four synergies, respectively. The differences between the *VAF*_*POST*_ obtained using generic synergies and the *VAF*_*PRE*_ obtained using subject-specific synergies, here used as reference value, were 7.3 ± 4.0%, 9.8 ± 5.6% and 8.6 ± 4.2% for AOA and 6.5 ± 4.0%, 10.7 ± 5.7% and 11.8 ± 7.5% for WOA, in the groups of two, three and four synergies, respectively. Using generic synergies was thus less accurate than using subject-specific synergies but the loss in accuracy was smaller for patients with less synergies. Nevertheless, the generic activation profiles (WOA) better explained pre- and post-treatment EMG than TD EMG for all groups while generic synergy weights (AOA) were only able to do so in the group with two and three synergies. More specifically, for the WOA *SPEC*_*PRE*_ was 23.1 ± 1.4%, 14.1 ± 3.0% and 3.9 ± 3.8% and *SPEC*_*POST*_ was 20.6 ± 3.7%, 11.7 ± 5.4% and 6.8 ± 6.8%. For the AOA, *SPEC*_*PRE*_ was 21.8% (SD 2.3%), 5.8% (SD 3.3%) and -1.4% (SD 7.2%) and *SPEC*_*POST*_ was 18.5% (SD 3.8%), 2.7% (SD 5.2%) and -0.6% (SD 3.9%).

### How well can subject-specific and generic synergies reconstruct activity of individual muscles?

**Fig 6:**
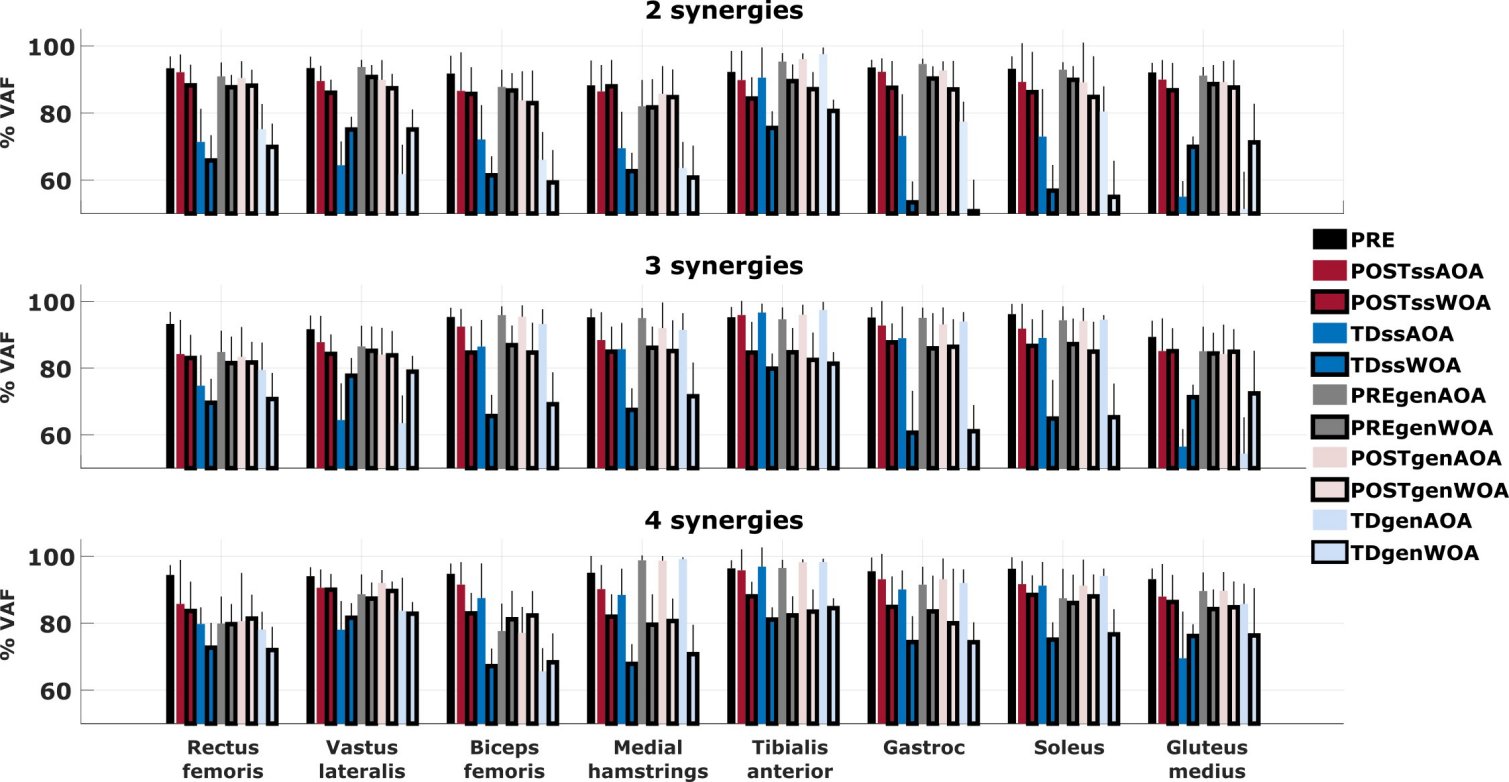
VAF computed independently for each of the analyzed muscles. Results from the EMG reconstructions using both subject-specific and generic synergies with both approaches, reported separately for each muscle. ss refers to the use of subject–specific synergies, gen to the use of generic synergies. AOA refers to the Activation Optinization Approach, WOA to the Weight Optimization Approach. Values for VAF_PRE_ are defined during synergy extraction with NNMF.

We further analyzed the performance of the reconstruction methods by separating the contributions of the eight muscles to the global VAF, for both subject-specific and generic synergies in both reconstruction approaches (Fig 6). On average, the difference between *VAF*_*POST*_ and *VAF*_*TD*_, defining the specificity of the reconstruction, for individual muscles was higher in the groups defined by a lower number of synergies. With respect to all the muscles considered in the analysis, the tibialis anterior showed higher values for the *VAF*_*TD*_, indicative of lower reconstruction specificity, in all the three groups. Especially, the specificity was lower for the AOA when using both the subject-specific and generic synergies.

For most muscles in the groups with two and four synergies, the values of *VAF*_*TD*_ were higher when using the AOA than when using the WOA, meaning that the use of pre-treatment synergy activations was more successful in distinguishing between the pathological and healthy activations. Vastus lateralis and gluteus medius, on the other hand, showed an opposite trend with high *SPEC*. Hence, for these two muscles, the constraints imposed by the pre-treatment synergies weights hindered the achievement of a non-pathological (TD) activation pattern.

### Do patient characteristics determine the performance of subject-specific synergies in reconstructing the post-treatment muscle activity?

**Fig 7:**
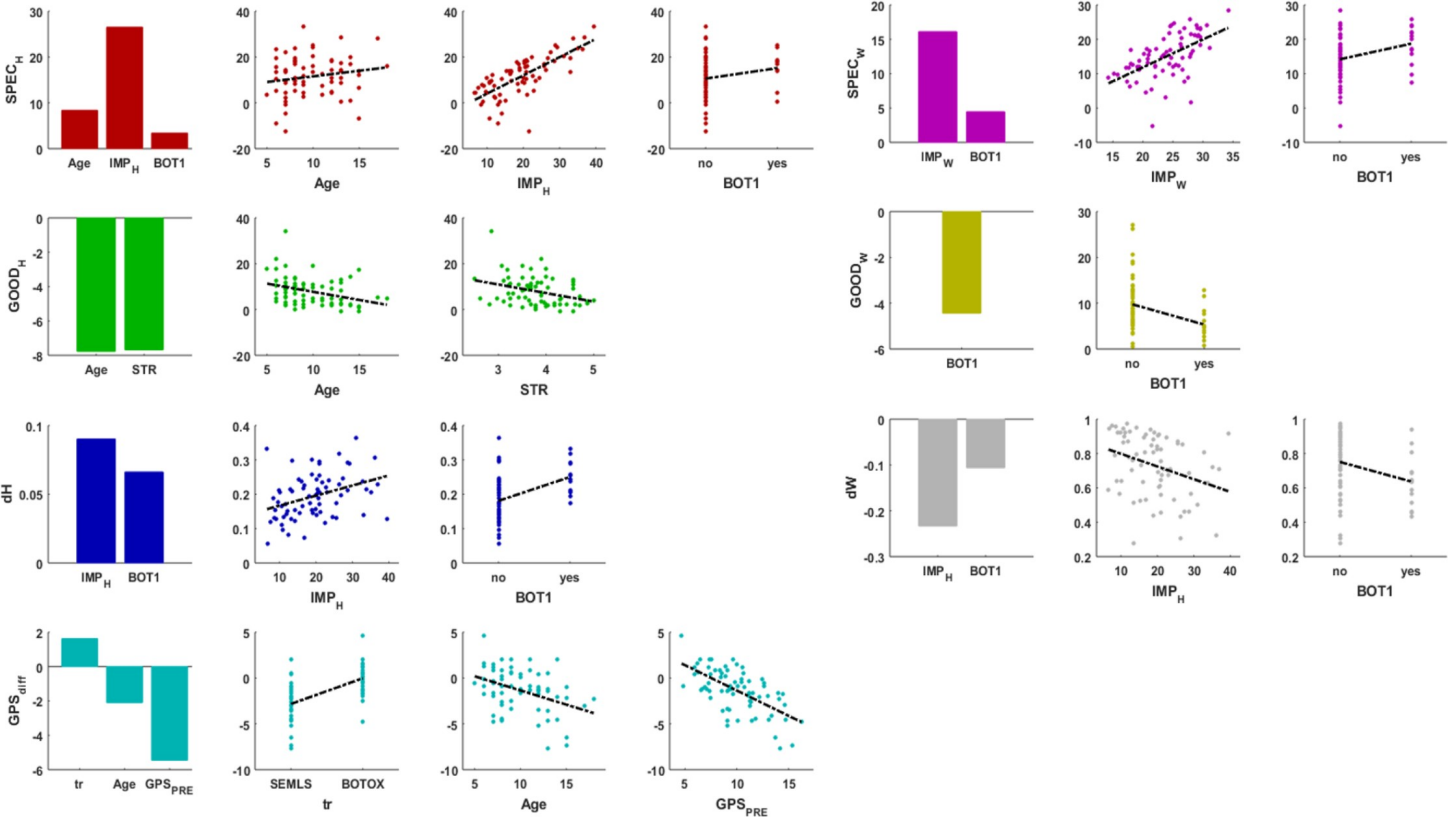
Results from the stepwise regression analysis. Results are reported for each of the dependent variables. The bar graphs report the effect sizes of the statistically significant independent variables of the fitted models. In the scatter plots the dependent variables are plotted as a function of each of the independent variables included in the model. SPEC specificity (%); GOOD goodness (%); IMP impairment of the subject pre-treatment (%); Age at time of surgery (years); STR average pre-treatment strength report; dW change in synergy weights expressed as the correlation coefficient between pre- and post-treatment; dH change in synergy activation profiles expressed as RMSE between pre- and post-treatment; GPS_PRE_ gait profile score pre-treatment (degrees); GPS_diff_ difference between GPS_PRE_ and GPS_POST_ (degrees); BOT1 rectus femoris injection (yes/no); tr treatment (BOTOX/SEMLS). Subscripts W and H indicate that the signal reconstruction was performed optimizing the weights or the activation profiles, respectively.

We performed a stepwise regression analysis to identify patient’s pre-treatment characteristics that explained (1) the performance of our reconstruction methods and (2) changes in synergy composition after treatment. The list of dependent and independent variables as well as the composition of the fitted models and the robustness of the fitting are listed in Table 2, whereas the effects of the variables included in the models are plotted in Fig 7. Reconstruction of post-treatment EMG based on pre-treatment synergy weights (AOA) was better, e.g. smaller differences between *VAF*_*PRE*_ and *VAF*_*POST*_, for older and stronger participants indicating that their muscle coordination patterns changed less after treatment (p < 0.001, R^2^ = 0.19). Independent of the approach used for signal reconstruction, e.g. using either the pre-treatment synergy weights or activation profiles for the reconstruction, the difference between *VAF*_*POST*_ and *VAF*_*TD*_ was larger when the difference between *VAF*_*PRE*_ and *VAF*_*TD*_ was larger, meaning that differences between the patient’s and TD control are preserved after treatment (AOA: p < 0.001, R^2^ = 0.60; WOA: p < 0.001, R^2^ = 0.39). Therefore, the use of subject-specific synergies to model post-operative motor control is especially important when the pre-treatment motor control is more impaired (motor control impairment is defined as *IMP = VAF*_*PRE*_*−VAF*_*TD*_). In addition, the difference between *VAF*_*POST*_ and *VAF*_*TD*_ is larger for patients who received BOTOX injection in their rectus femoris than for those children who received BOTOX in other muscles but not rectus, indicating that patients requiring BOTOX in the rectus femoris had more impaired motor control. When using the synergy weights for the reconstruction, this difference also increases with age. The post-treatment muscle synergy weight vectors (p = 0.0018, R^2^ = 0.16) and activation profiles (p < 0.001, R^2^ = 0.30) differed more from the pre-treatment ones in patients with greater control impairments and patients that received BOTOX injections in their rectus femoris. The change in deviation of the kinematics from TD, characterized by the gait profile score (*GPS*_*diff*_), was not predicted by any synergy related quantity but depended on *GPS*_*pre*_, with better pre-treatment performance associated with smaller improvements or even worsening after the treatment (p < 0.001, R^2^ = 0.61).

## Discussion

BOTOX and SEMLS treatments had little influence on the motor control of patients with CP. Therefore, subject-specific muscle synergies computed from pre-treatment EMG data were able to reconstruct the post-treatment EMG signals, explaining on average more than 85% of their variability. Interestingly, this was true for both analyzed treatments, highlighting the resilience of impaired motor control to therapeutically induced changes in the mechanics of the musculoskeletal system. These findings are in agreement with previous studies reporting little change in muscle activity and muscle synergies after orthopedic treatments in patients with CP [26,27]. In addition, this study further contributes to the field by evaluating the use of a pre-treatment model of motor control in predictive simulations of the patient’s post-treatment condition. In this case, the purpose of muscle synergies is to constrain the potential muscle activations that drive the musculoskeletal model, in order to be representative of the ones observed in the patient.

Although the VAF of the post-treatment EMG explained by subject-specific synergies was high, it was about 8% lower than the VAF in the pre-treatment condition. The motor control of the patients is thus susceptible to some changes between the pre- and post-treatment conditions. Part of this change was found to be dependent on the age at which the patients underwent the treatment. It is likely that, despite the impaired motor control, younger patients can more easily adapt to changes in the musculoskeletal system introduced by the treatment.

Interestingly, the two proposed EMG reconstruction methods (keeping either the synergy weights or activation profiles constant) gave similar results when applied to the post-treatment data of the patients. It seems likely that, in the post-treatment period, both the composition and the activation of the muscle synergies go through some minor changes and, by selecting one of the two reconstruction methods, i.e. optimizing either the activations or the compositions, it was possible to only partially reproduced the adaptation strategy of the patients’ motor control.

Furthermore, patients with a more impaired pre-treatment motor control showed more changes in both synergy weights and activation profiles. However, the low R^2^ values for most of the fitted models suggest that there are important factors not included in our analysis that influence the observed changes in motor control. Muscle synergies in children with CP have shown a good repeatability between measurement sessions, especially when computed over multiple gait cycles [38,39]. However, some variability between measurements was present and, although small, could have affected our findings. Furthermore, another confounding factor could be the contributions of spasticity to the EMG signals of some muscles. Spastic muscles exhibit exaggerated stretch reflexes, which contribute to the observed EMG signal. However, this contribution is dependent on the state of the muscle (elongation, force and/or their derivatives) [40] and can be affected by the administered treatment. Thus leading to EMG signal differences even when no change in motor control is present. Removing spasticity contributions from the EMG signals before computing muscle synergies might improve the interpretation and utilization of synergies. Future studies need to try to investigate this hypothesis.

Subject-specific pre-treatment synergies capture the specific impairments of the motor control in CP patients, as they were less successful in explaining muscle activity observed in an unimpaired population than the muscle activity recorded after the treatment, especially in patients with a lower number of synergies. This makes them a useful tool to describe motor control in simulations of CP gait. Generic synergies did not perform as well as subject-specific synergies in explaining the patients’ muscle activations. However, for patients with a low selectivity (small number of synergies), they were able to account for more than 88% of pre-treatment variability and more than 85% of post-treatment variability. For these patients, generic synergies were also able to capture features of the motor impairment that are shared between patients since they had a better performance when reconstructing pathological activations than when reconstructing typically developed ones, with an average difference of 21% VAF.

The analyses performed in this study were based on surface EMG signals collected from eight major muscles per leg. Using a different set of muscles or a different type of EMG signal acquisition, the results may differ. Keeping this limitation in mind, from our results we can conclude that muscle synergies computed before the treatment are a good model of the post-treatment motor control in patients with CP, since the motor control in these patients remains mostly unchanged after the received treatment, as reflected by the limited decrease in signal reconstruction accuracy. In addition, it is possible to use generic synergies as template for pathological CP motor control, as they are able to produce activations more similar to those measured in patients than in typically developing children. Both pre-treatment synergy weights and activations are suitable for this purpose and they could be used to model the post-treatment motor control at the light of two different hypothesis of motor control adaptation.

These results support the feasibility of using information about the motor control collected before a treatment to run computational simulations depicting the post-treatment condition when treating patients with CP.

## Supporting information

S1 Appendix Overview patients and administered treatments. (XLSX)
